# A multi-level examination of school programs, policies and resources associated with physical activity among elementary school youth in the PLAY-ON study

**DOI:** 10.1186/1479-5868-7-6

**Published:** 2010-01-25

**Authors:** Scott T Leatherdale, Steve Manske, Guy Faulkner, Kelly Arbour, Chad Bredin

**Affiliations:** 1 Department of Population Studies and Surveillance, Cancer Care Ontario, Toronto, Ontario, Canada; 2 Propel Centre for Population Health Impact, Canadian Cancer Society and the University of Waterloo, Waterloo, Ontario, Canada; 3 Dalla Lana School of Public Health, University of Toronto, Toronto, Ontario, Canada; 4 Faculty of Physical Education and Health, University of Toronto, Toronto, Ontario, Canada

## Abstract

**Background:**

Given the decline in physical activity (PA) levels among youth populations it is vital to understand the factors that are associated with PA in order to inform the development of new prevention programs. Many studies have examined individual characteristics associated with PA among youth yet few have studied the relationship between the school environment and PA despite knowing that there is variability in student PA levels across schools.

**Methods:**

Using multi-level logistic regression analyses we explored the school- and student-level characteristics associated with PA using data from 2,379 grade 5 to 8 students attending 30 elementary schools in Ontario, Canada as part of the PLAY-Ontario study.

**Results:**

Findings indicate that there was significant between-school random variation for being moderately and highly active; school-level differences accounted for 4.8% of the variability in the odds of being moderately active and 7.3% of the variability in the odds of being highly active. Students were more likely to be moderately active if they attended a school that used PA as a reward and not as discipline, and students were more likely to be highly active if they attended a school with established community partnerships. Important student characteristics included screen time sedentary behaviour, participating in team sports, and having active friends.

**Conclusion:**

Future research should evaluate if the optimal population level impact for school-based PA promotion programming might be achieved most economically if intervention selectively targeted the schools that are putting students at the greatest risk for inactivity.

## Introduction

Participation in physical activity (PA) is an integral component of a healthy lifestyle as it is associated with a number of positive health benefits, such as reduced risk of several chronic diseases and improved cardiorespiratory fitness [1]. Given the decline in PA levels among youth populations [2], it is vital to understand the factors that are associated with PA in order to inform the development of new prevention programs. This is critical, as increasing PA among youth offers great potential to reduce the future health burden at the population-level [3].

Many studies have examined individual characteristics associated with PA among youth [4]. For instance, research has identified that youth are more likely to be active if they are male [4,5], if they participate in team sports [6,7], if they spend less time in sedentary screen-time behaviours [5,8], if they are a healthy body weight [5,9], and if they have friends who are active [5]. However, there is also a need to take a broader ecological approach that not only considers characteristics of the individual, but also the influential contexts (e.g., school environment) in which that individual is situated [10]. According to ecological theory [10], due to the dynamic nature of interactions between factors at various contextual levels, it would not be individual characteristics or school characteristics alone that influence a particular behaviour, but rather the combination and interaction of these different factors that result in behaviour such as PA. By using an ecological approach, researchers can examine individual characteristics and different contextual influences simultaneously, creating a better understanding of the determinants of behaviour.

Examining the relationship between the school environment and PA is important since Canadian youth spend ~25 hours each week in school throughout the school year and because school-based PA can account for up to 40% of the total activity among youth populations [11]. Consistent with the tenets of ecological theory [10], there is now recognition of the need to adopt a broader 'whole school' approach in promoting PA that seeks to identify the influential aspects of the school environment, in terms of the physical environment, policies or practices, so that they can be modified [12]. While we know that there is variability in student PA levels across schools and that different school characteristics provide youth with different opportunities to engage in PA [13-15], research has yet to examine how school environments promote or inhibit PA within this type of 'whole school' framework. For instance, although Sallis and colleagues [14] identified that students were more likely to be active if they attended a school where there had been recent improvements made to their school environment (e.g., building basketball courts), they did not consider this within the broader context of different PA curriculum, policies, programs, or resources within the school environment; valuable contextual insight for practitioners. Considering that data on multiple PA related programs, policies and resources in schools are typically not systematically collected and examined [16], few researchers have had the data required for using a 'whole school' approach within their research.

In order to appropriately tailor and target PA interventions so that they are most likely to have impact, research is needed that simultaneously examines contextual and individual factors associated with PA. It has been suggested that this is particularly important within elementary school settings since much of the available evidence pertaining to school characteristics is garnered from secondary school settings [17]. The purpose of the present study is to better understand the school- and student-level characteristics associated with PA among elementary school youth. Such insight would be valuable for informing the creation of future school-based PA initiatives.

## Methods

### Design

This cross-sectional study used self-reported data collected in 2007-2008 from a convenience sample of grade 5 to 8 students attending 30 elementary schools in Ontario, Canada as part of the PLAY-Ontario (Play-On) study. Student-level data were collected from consenting students using the Physical Activity Module (PAM) of the School Health Action, Planning and Evaluation System (SHAPES). The PAM asks students about physical activity, height and weight, sedentary behaviours, correlates of physical activity, and participation in teams and sporting activities at school. Validity testing [18] has demonstrated significant criterion validity based on Spearman correlations for our self-reported measures of height (r = 0.97, p < .001), weight (r = 0.98, p < .001), and PA (r = 0.44, p < .01). Additional details about PLAY-On are available online http://www.shapes.uwaterloo.ca/projects/PLAYON, and additional details about SHAPES and the PAM measures and their psychometric properties are available in print [18,19].

School-level data were collected using the physical activity categories of the elementary school version of the School Health Environment Survey (SHES) [20]. The SHES is a tool that is designed to assess programs, activities, committees, facilities and guidelines surrounding physical activity and healthy eating in the school environment. These four physical activity categories are aligned with the Government of Ontario's Foundations for a Healthy School [21]. Additional details about the SHES measures and assessment categories are available in print [20] and online http://www.shapes.uwaterloo.ca/SHES.

### Data Collection

All students at the participating schools were eligible to participate. Prior to participating in the study, active consent from parents was required and at any time students were able to decline participation. Eligible students completed the PAM during class time. At each participating school, the administrator(s) most knowledgeable about the school's programs, policies and resources was asked to complete the SHES survey. The University of Waterloo Office of Research Ethics and appropriate School Board Ethics committees approved the study procedures.

### Participants

Of the 4,838 students enrolled in grades 5 to 8 at the 30 participating elementary schools, 50.6% (n = 2,449) participated in the survey; missing respondents resulted from parent/student refusal and absenteeism on the day of the survey. This distribution is consistent with a previous active consent study examining obesity and physical activity among Canadian elementary students [22]. Since 2.8% (n = 70) of participating students did not provide PA data, the final sample was 2,379 students. The SHES survey was completed by all 30 elementary schools.

### Measures

#### Outcomes - Physical Activity

Physical activity level was based on kilocalories per kilogram of body weight per day (KKD). Using validated measures [18], students were asked how many minutes of vigorous physical activity (VPA) and moderate physical activity (MPA) they engaged in on each of the last seven days. The average KKD expended in VPA and MPA were calculated as: KKD = [(Hours of VPA*6 MET)+(Hours of MPA*3 MET)]/7 days

Note: assuming that the standard metabolic equivalent (MET, a unit used to estimate the amount of oxygen used by the body during physical active) for VPA was 6 and MPA is 3 as per CDC guidelines http://www.cdc.gov/nccdphp/dnpa/physical/terms.

The SHAPES measures are valid for differentiating students who report less time engaging in PA from those who report more time engaged in PA [18]. Hence, within our sample, students more than one standard deviation (≤ 16^th ^percentile) below the sample mean for KKD were classified as low active, students more than one standard deviation (≥ 84^th ^percentile) above the sample mean for KKD were classified as highly active; all others were classified as moderately active.

#### Student-Level Correlates

Body Mass Index (BMI) was calculated using validated [18] self-report measures of weight (kg) and height (m) (BMI = kg/m^2^). BMI categories were consistent with CDC guidelines and growth charts [23] as described elsewhere [8]. For the multivariate analyses, students classified as overweight or obese were collapsed into one category (*overweight*) to represent all youth who may be at-risk for morbidity associated with being overweight and to ensure there was sufficient power for the multi-level analyses. The measures for sedentary behaviour, sports participation, and social influences were consistent with previous research [6,9]. Respondents reported the number of hours for each day of the week that they spent watching TV/movies, playing video/computer games, surfing the internet, instant messaging or talking on the phone. We calculated the average screen time per day based on the average time reported over the previous week, and grouped responses into three categories (<1 hour/day, 1 to 3 hours per day, >3 hours/day). Respondents also reported whether or not they participate in varsity or team sports at school (yes/no), whether or not they participate in intramural or house league sports at school (yes/no), whether or not they participate in league or team sports outside of school (yes/no), and how many of their close friends are physically active (0-5).

#### School-Level Characteristics

Consistent with the four components that form the basis of the Foundations for a Healthy School (FHS) according to the Ontario Ministry of Education [21], the SHES physical activity tool measured indicators associated with: *Healthy Physical Environment *(availability of, access to, and adequacy in meeting student needs for, indoor and outdoor facilities, equipment and resources for safe, quality physical activity on or near school grounds, both during and outside of school hours); *Instruction and Programs *(availability, delivery and characteristics of curricular physical education, extracurricular physical activity programs, and active transportation to school, including barriers to implementing such programs); *Supportive Social Environment *(characteristics of the school's social environment that predispose, reinforce and enable enjoyable, lifelong participation in physical activity or that hinder such activities); and *Community Partnerships *(the accessibility and availability of support services for physical activity which may include partnerships with public health units and community based services and resources). Each indicator was assigned a classification by the research team based on the corresponding phase of implementation in the Healthy School Continuum as outlined by the Joint Consortium on School Health [24]: *Initiation *(falls short or exhibits extensive room for improvement in meeting the recommendations related to school capacity for physical activity); *Action *(meets the recommendations in several, but not all areas related to school capacity for physical activity, exhibits some room for improvement); *Maintenance *(consistently meets or exceeds the recommendations related to school capacity for physical activity, encouraged to maintain the current level of commitment to supporting physical activity at school). Each of the four FHS components was also assigned an 'overall' phase classification based on the combined responses to component indicators. The assessment schemes for the SHES measures were developed based on the current research literature, Government of Ontario guidelines, and input from experts in the domains of healthy eating and physical activity in schools [19].

#### High-Risk vs. Low-Risk Schools

A school was considered 'high-risk' if the overall indicator for three of the four FHS components was in the initiation phase, whereas a school was considered 'low-risk' if all four indicators for the overall FHS components were in the action or maintenance phase.

#### Analyses

Using student-level data, we calculated the prevalence of physical activity, weight status, screen time, and the social influences and team sport correlates by sex. Using school-level data, we calculated the prevalence of the indicators for healthy school environment, instruction and programs, supportive social environment, and community partnerships by the phase of implementation. A chi-square analysis was used to examine differences in physical activity between high-risk and low risk schools. To understand the student- and school-level factors associated with PA, we performed two series of multi-level logistic regression analyses to examine characteristics associated with being (1) moderately active versus low active, and (2) highly active versus low active. Consistent with other studies [25], each analysis used a stepped modelling procedure. Step 1 examined if the differences in being moderately active or highly active were random or fixed across schools. The school-level variance term from Step 1 (σ^2^_*μ0*_) was used to calculate the intraclass correlation (ICC) for binary outcomes [26]:

In Step 2, a series of univariate analyses were used as exploratory analyses to examine if the implementation phase score for each of the FHS indicators were associated with being moderately active or highly active. Only significant school-level variables (p < 0.05) were retained for further analyses. We did not perform univariate analyses with the student characteristics gender, BMI, sedentary behaviour, sports participation and social influences since the published literature had previously demonstrated that they were associated with PA [4-9]. In Step 3, multivariate models were developed to examine how the student characteristics and the significant school characteristics identified in Step 2 were associated with being moderately active (Model 1) and highly active (Model 2). After the final models were developed, we explored contextual interactions between all of the significant (p < 0.05) school and student characteristics to examine potential moderating effects between school and student characteristics using the interaction function in ML*wiN*. Statistical analyses were conducted with ML*wiN *Version 2.02 [27].

## Results

### Student Characteristics

The sample was 47.3% (n = 1,126) male and 52.7% (n = 1,253) female. The average age was 11.6 (± 1.1) years; the age distribution was not significantly different between males and females (χ^2 ^= 5.54, *df *= 5, p = 0.353). Overall, 16.4% of students were classified as low active, 67.2% were classified as moderately active, and 16.4% were classified as highly active. Males were more likely to be highly active compared to females (χ^2 ^= 15.05, *df *= 2, p < .001). The mean BMI among males was 19.5 (± 3.8) kg/m^2 ^and 19.1 (± 4.1) kg/m^2 ^among females. Males were more likely to be overweight or obese compared to females, although females were more likely to have missing BMI data compared to males (χ^2 ^= 12.46, *df *= 3, p < .01). The majority of students (63.0%) reported 1 to 3 hours of screen time per day. Males were more likely than females to report spending 3 or more hours per day on screen time activities (χ^2 ^= 29.62, *df *= 2, p < .01). Very few students reported that they have less than three close friends who are physically active (9.7%). Males were more likely than females to report having five close friends who are physically active (χ^2 ^= 45.88, *df *= 5, p < .001). The majority of students also reported that they participate in intramural sports at school (64.1%), varsity sports at school (65.4%), or league sports outside of school (72.3%). There were no sex differences in the prevalence of students participating in varsity or intramural team sports at school, whereas males were more likely than females to participate in league sports outside of school (χ^2 ^= 9.59, *df *= 1, p < .01). Demographic characteristics of students by sex are presented in Table 1.

**Table 1:** Descriptive statistics for youth in grades 5 to 8 by sex (Ontario, Canada)

	Male (n = 1,126)		Female (n = 1,253)		Chi-Square
*Student-Level Characteristics*	%	(n)^a^	%	(n)^a^	
**Physical activity level**					
Highly active	19.6	(220)	13.6	(171)	χ^2 ^= 15.05, *df *= 2
Moderately active	64.9	(731)	69.3	(868)	p < .001
Low active	15.5	(175)	17.1	(214)	
**Weight status ^b^**					
Normal weight	39.5	(444)	36.3	(455)	χ^2 ^= 43.24, *df *= 3,
Overweight	10.1	(114)	5.6	(70)	p < .001
Obese	6.0	(68)	3.2	(40)	
Missing	41.1	(462)	52.0	(651)	
**Screen time per day**					
<1 hour per day	18.6	(208)	24.9	(308)	χ^2 ^= 29.62, *df *= 2,
1 to 3 hours per day	62.6	(699)	63.3	(784)	p < .001
>3 hours per day	18.8	(210)	11.8	(146)	
**Number of close friends who are physically active**					
None	0.5	(6)	0.3	(4)	χ^2 ^= 45.88, *df *= 5,
1	2.5	(27)	2.5	(31)	p < .001
2	6.6	(73)	6.9	(85)	
3	14.4	(159)	25.2	(312)	
4	26.4	(291)	24.8	(307)	
5	49.6	(546)	40.3	(500)	
**Intramural sports at school**					
Does not participate	37.2	(400)	34.8	(415)	χ^2 ^= 1.43, *df *= 1,
Does participate	62.8	(676)	65.2	(779)	p = .231
**Varsity sports at school**					
Does not participate	33.7	(364)	35.4	(428)	χ^2 ^= 0.75, *df *= 1,
Does participate	66.3	(717)	64.6	(781)	p = 385
**League sports outside of school**					
Does not participate	24.7	(269)	30.5	(371)	χ^2 ^= 9.59, *df *= 1,
Does participate	75.3	(821)	69.5	(847)	p < .01
**Grade**					
5	23.2	(261)	25.5	(320)	χ^2 ^= 5.19, *df *= 3,
6	26.6	(300)	25.9	(324)	p = .158
7	25.4	(286)	27.2	(341)	
8	24.8	(279)	21.4	(268)	

^a ^Numbers may not add to total because of missing values

^b ^BMI values used to determine weight status have been adjusted for age and gender

### School Characteristics

The mean prevalence of moderately active students at a school was 66.6% (range, 53.6% to 82.8%) and the mean prevalence of highly active students at a school was 16.6% (range, 4.2% to 30.3%). The majority of schools were in the action phase for the overall indicator scores for Healthy Physical Environment (66.7%) and Supportive Social Environment (66.7%) and the maintenance phase for the overall score for Community Partnerships (56.6%). Conversely, the majority of schools were in the initiation phase for the overall score for Instruction and Programs (73.3%). None of the schools were in the maintenance phase for the overall scores for Healthy Physical Environment, Instruction and Programs, and Supportive Social Environment. Within each of the four FHS components, there was substantial variability across schools in relation to the individual indicators measured. The descriptive statistics for school-level indicators are presented in Table 2.

**Table 2:** Descriptive statistics for the school-level indicators among the 30 elementary schools (Ontario, Canada)

	Initiation	Action	Maintenance
	% (n)	% (n)	% (n)
**Healthy Physical Environment**			
Student access to a variety of facilities on and off school grounds during school hours	3.3 (1)	40.0 (12)	56.7 (17)
Availability of physical activities during inclement weather	53.3 (16)	40.0 (12)	6.7 (2)
Student access to facilities and equipment outside of school hours	33.3 (10)	63.4 (19)	3.3 (1)
Support for active transportation to/from school	23.3 (7)	46.7 (14)	30.0 (9)
***Overall score for this indicator***	***33.3 (10)***	***66.7 (20)***	**-**
**Instruction and Programs**			
Implementation of daily PA	-	80.0 (24)	20.0 (6)
Time spent per week engaged in PA during physical education classes	93.4 (28)	3.3 (1)	3.3 (1)
Classes taught by a qualified physical education specialist	86.7 (26)	13.3 (4)	-
Availability and use of intramural/club activities	80.0 (24)	13.3 (4)	6.7 (2)
Consistency of intramural programming across grade divisions and seasons	36.7 (11)	43.3 (13)	20.0 (6)
Availability and use of interschool programs	53.4 (16)	43.3 (13)	3.3 (1)
Consistency of interschool programming across seasons	16.7 (5)	-	83.3 (25)
***Overall score for this indicator***	***73.3 (22)***	***26.7 (8)***	-
**Supportive Social Environment**			
Emphasis placed on maximizing participation in PA through school programs	10.0 (3)	23.3 (7)	66.7 (20)
Incorporation of PA into other school subjects	20.0 (6)	63.3 (19)	16.7 (5)
Special recognition of students who participate in school physical activities	10.0 (3)	20.0 (6)	70.0 (21)
Formal collection of suggestions from the school community about PA at school	60.0 (18)	30.0 (9)	10.0 (3)
Promotion of PA programs and events for students, families and school staff	23.3 (7)	30.0 (9)	46.7 (14)
Use of PA as a reward, not as discipline	40.0 (12)	40.0 (12)	20.0 (6)
Presence of written policies/practices for PA	20.0 (6)	53.3 (16)	26.7 (8)
***Overall score for this indicator***	***33.3 (10)***	***66.7 (20)***	**-**
**Community Partnerships**			
Support available for staff involved with PA	-	30.0 (9)	70.0 (21)
Connection to community resources	20.0 (6)	13.3 (4)	66.7 (20)
***Overall score for this indicator***	***16.7 (5)***	***26.7 (8)***	***56.6 (17)***

PA = physical activity

### High Risk vs. Low Risk Schools

Based on the distribution of the school-level indicators, a total of four schools were classified as low risk for physical inactivity and six schools were classified as high risk for physical inactivity. All of the low risk schools in this sample were in the action phase for Healthy Physical Environment, Instructions and Programs, and Supportive Social Environment and the maintenance phase for Community Partnerships, whereas all of the high risk schools were in the initiation phase for Healthy Physical Environment; high risk schools varied with respect to being in the initiation and action phases for the other three FHS components. Students attending a low risk school were more likely to be highly active than students attending a high risk school (χ^2 ^= 6.17, *df *= 1, p < .05). Similarly, students attending a low risk school were even more likely to be moderately active than students attending a high risk school (χ^2 ^= 6.71, *df *= 1, p < .01).

### School Characteristics Associated with being Moderately Active or Highly Active

There was significant between-school random variation identified for being moderately active [σ^2^_μ0 _= 0.166(0.043), p < 0.001] and being highly active [σ^2^_μ0 _= 0.259(0.067), p < 0.001]. This suggests that school-level differences accounted for 4.8% of the variability in the odds of being moderately active and 7.3% of the variability in the odds of being highly active. Table 3 presents the results of the univariate analyses examining the associations between student physical activity and the FHS school-level indicators.

**Table 3:** Multi-level logistic regression analyses examining univariate associations between the school-level indicators and physical activity among youth in grades 5 to 8 (Ontario, Canada)

		Model 1 **Estimate (SE)**^§^	Model 2 **Estimate (SE)**^§^
**Healthy Physical Environment**			
Student access to a variety of facilities on and off school grounds during school hours^†^	Action Maintenance	-0.01 (0.17) -0.02 (0.20)	-0.17 (0.19) 0.14 (0.23)
Availability of physical activities during inclement weather^†^	Action Maintenance	0.18 (0.14) 0.36 (0.25)	0.25 (0.22) 0.52 (0.40)
Student access to facilities and equipment outside of school hours^†^	Action Maintenance	-0.04 (0.23) 0.23 (0.50)	-0.10 (0.27) 0.68 (0.28)*
Support for active transportation to and from school^†^	Action Maintenance	-0.18 (0.23) -0.10 (0.24)	-0.08 (0.27) 0.01 (0.28)
***Overall score for this indicator***^†^	Action	-0.15 (0.16)	-0.15 (0.20)
**Instruction and Programs**			
Implementation of daily PA^‡^	Maintenance	0.26 (0.19)	0.13 (0.26)
Time spent per week engaged in PA during physical education classes^†^	Action Maintenance	-0.74 (0.39) 0.01 (0.42)	-0.47 (0.53) -0.60 (0.57)
Classes taught by physical education specialist^†^	Action	0.26 (0.21)	0.28 (0.28)
Availability and use of intramural/club activities^†^	Action Maintenance	0.06 (0.16) 0.04 (0.36)	0.30 (0.25) 0.28 (0.51)
Consistency of intramural programming across grade divisions and seasons^†^	Action Maintenance	-0.06 (0.17) 0.07 (0.23)	-0.04 (0.22) 0.19 (0.29)
Availability and use of interschool programs^†^	Action Maintenance	0.16 (0.17) -0.66 (0.42)	-0.06 (0.22) -0.37 (0.54)
Interschool programs across seasons^†^	Maintenance	-0.05 (0.22)	-0.31 (0.28)
***Overall score for this indicator***^†^	Action	0.10 (0.17)	-0.34 (0.20)
**Supportive Social Environment**			
Emphasis placed on maximizing participation in PA through school programs^†^	Action Maintenance	0.08 (0.29) 0.24 (0.28)	-0.31 (0.34) -0.44 (0.33)
Incorporation of PA into other school subjects^†^	Action Maintenance	-0.02 (0.22) -0.49 (0.29)	0.36 (0.35) 0.01 (0.47)
Special recognition of students who participate in school physical activities^†^	Action Maintenance	-0.63 (0.26)* -0.46 (0.22)*	-0.36 (0.42) -0.67 (0.30)*
Formal collection of suggestions from the school community about PA at school^†^	Action Maintenance	-0.36 (0.19) 0.43 (0.35)	-0.14 (0.27) 0.99 (0.55)
Promotion of PA programs and events for students, families and school staff^†^	Action Maintenance	-0.19 (0.18) 0.22 (0.22)	0.06 (0.30) 0.07 (0.36)
Use of PA as a reward, not as discipline^†^	Action Maintenance	0.34 (0.15)* 0.64 (0.22)*	0.28 (0.28) 0.13 (0.36)
Presence of written policies or practices that support PA^†^	Action Maintenance	0.29 (0.18) 0.33 (0.21)	0.36 (0.29) 0.62 (0.34)
***Overall score for this indicator***^†^	Action	0.20 (0.16)	0.30 (0.19)
**Community Partnerships**			
Support available for staff involved with PA^‡^	Maintenance	-0.09 (0.21)	0.01 (0.24)
Connection to community resources^†^	Action Maintenance	0.14 (0.30) 0.24 (0.24)	0.08 (0.34) 0.48 (0.28)
***Overall score for this indicator***^†^	Action Maintenance	-0.18 (0.23) 0.08 (0.21)	0.05 (0.26) 0.51 (0.23)*

Note: ^§ ^A series of univariate analyses, ^† ^Reference group is Initiation, ^‡ ^Reference group is Action

Model 1: 1 = Moderately Active (n = 1,599), 0 = Low Active (n = 389); Model 2: 1 = Highly Active (n = 391), 0 = Low Active (n = 389) * p < .05

Univariate analyses identified that Supportive Social Environment was the only FHS category that had any school-level indicators significantly associated with a student being moderately active. A student was less likely to be moderately active if he/she attended a school that was in the action or maintenance phase for the indicator *Special recognition of students who participate in school physical activities *[β = -0.63(0.26) and β = -0.46(0.22) respectively]. Conversely, a student was more likely to be moderately active if he/she attended a school that was in the action or maintenance phase for the indicator *Use of PA as a reward, not as discipline *[β = 0.34(0.15) and β = 0.64(0.22) respectively]. None of the indicators within the other three FHS categories or the overall FHS component scores were significantly associated with being moderately active.

Univariate analyses identified that indicators from three of the FHS categories were significantly associated with a student being classified as highly active. In the Healthy Physical Environment category, a student was more likely to be highly active if he/she attended a school that was in the maintenance phase for the indicator *Student access to facilities and equipment outside of school hours *[β = 0.68(0.28)]. In the Supportive Social Environment category, a student was less likely to be highly active if he/she attended a school that was in the maintenance phase for the indicator *Special recognition of students who participate in school physical activities *[β = -0.67(0.30)]. In the Community Partnerships category, a student was more likely to be highly active if he/she attended a school that was in the maintenance phase for the overall score for this category compared to a student attending a school that was in the initiation phase for the overall score [β = 0.51(0.23)]. The only FHS category which did not have any indicators significantly associated with a student being highly active was Instruction and Programs.

### School- and Student-level Characteristics Associated with being Moderately Active

The adjusted odds ratios are presented in Table 4 (Model 1). A student with three or more close friends who are active was more likely to be moderately active than a student with less than three friends who are active (OR 2.36, 95%CI 1.67 to 3.32). A student who participated in intramural sports at school was more likely to be moderately active than a student who did not participate in intramural sports at school (OR 1.80, 95%CI 1.34 to 2.41). A student who participated in league sports outside of school was also more likely to be moderately active than a student who did not participate in league sports outside of school (OR 2.18, 95%CI 1.67 to 2.85). Conversely, a student who reported three or more hours of screen time per day was less likely to be moderately active than a student who reported less than one hour of screen time per day (OR 0.41, 95%CI 0.20 to 0.82). Weight status, participation in varsity sports, and sex were not associated with being moderately active. The only school characteristic associated with being moderately active in the final model was *Use of PA as a reward, not as discipline*. If a student attended a school that was in the action or maintenance phase for the indicator *Use of PA as a reward, not as discipline*, he/she was more likely to be moderately active than a similar student attending a school that was in the initiation phase for this indicator (OR 1.43, 95%CI 1.03 to 1.98 and OR 1.57, 95%CI 1.06 to 2.32 respectively). There were no significant contextual interactions identified.

**Table 4:** Odds ratios for school- and student-level factors associated with being moderately active or highly active among youth in grades 5 to 8 (Ontario, Canada)

	Adjusted Odds Ratio^§ ^(95% CI)	
	Model 1	Model 2
*Student-Level Characteristics*	Moderately Active vs. Low Active	Highly Active vs. Low Active
**Screen time per day**		
<1 hour per day	1.00	1.00
1 to 3 hours per day	0.91 (0.57, 1.46)	0.94 (0.60, 1.45)
>3 hours per day	0.41 (0.20, 0.82)*	0.64 (0.43, 0.97)*
**Number of close friends who are physically active**		
None to 2 friends	1.00	1.00
3 or more	2.36 (1.67, 3.32)**	4.48 (2.29, 8.67)**
**Intramural sports at school**		
Does not participate	1.00	1.00
Does participate	1.80 (1.34, 2.41)**	3.15 (2.04, 4.86)**
**Varsity sports at school**		
Does not participate	1.00	1.00
Does participate	0.99 (0.73, 1.32)	1.37 (0.87, 2.15)
**League sports outside of school**		
Does not participate	1.00	1.00
Does participate	2.18 (1.67, 2.85)**	3.86 (2.52, 5.91)**
**Weight status ^a^**		
Normal weight	1.00	1.00
Overweight	0.97 (0.63, 1.50)	0.65 (0.36,1.16)
Missing	0.80 (0.61, 1.04)	0.46 (0.31, 0.68)***
**Sex**		
Female	1.00	1.00
Male	1.06 (0.82, 1.36)	1.60 (1.11, 2.30)**
***School-Level Characteristics***		
**Special recognition of students who participate in school physical activities**		
**Use of PA as a reward, not as discipline**		
Initiation	1.00	-
Action	1.43 (1.03, 1.98)*	
Maintenance	1.57 (1.06, 2.32)*	
**Community partnerships (*Overall score*)**		
Initiation	-	1.00
Action		2.79 (1.39, 5.59)**
Maintenance		2.81 (1.41, 5.63)**

Note: §Odds ratios adjusted for all other variables in the table and controlling for grade and the school level characteristics 'Special recognition of students who participate in school physical activities' and 'Student access to facilities and equipment outside of school hours'.

^a ^BMI values used to determine weight status have been adjusted for age and gender

Model 1: 1 = Moderately Active (n = 1,599), 0 = Low Active (n = 389)

Model 2: 1 = Highly Active (n = 391), 0 = Low Active (n = 389)

* p < .05 **p < .01

### School- and Student-level Characteristics Associated with being Highly Active

The adjusted odds ratios are presented in Table 4 (Model 2). Male students were more likely to be highly active than female students (OR 1.60, 95%CI 1.11 to 2.30). A student with three or more close friends who are active was more likely to be highly active than a student with less than three friends who are active (OR 4.48, 95%CI 2.29 to 8.67). A student who participated in intramural sports at school was more likely to be highly active than a student who did not participate in intramural sports at school (OR 3.15, 95%CI 2.04 to 4.86). A student who participated in league sports outside of school was also more likely to be highly active than a student who did not participate in league sports outside of school (OR 3.86, 95%CI 2.52 to 5.91). Conversely, a student who reported three or more hours of screen time per day was less likely to be highly active than a student who reported less than one hour of screen time per day (OR 0.64, 95%CI 0.43 to 0.97). A student who did not report his/her weight status was also less likely to be highly active than a normal weight student (OR 0.46, 95%CI 0.31, 0.68). Participation in varsity sports was not associated with being highly active. The only school characteristic associated with being highly active in the final model was the overall category score for *Community Partnerships*. If a student attended a school that was in the action or maintenance phase for the overall score for *Community Partnerships*, he/she was more than twice as likely to be highly active than a similar student attending a school that was in the initiation phase for this overall category score (OR 2.78, 95%CI 1.39 to 5.59 and OR 2.81, 95%CI 1.41 to 5.63 respectively).

**Figure 1:**
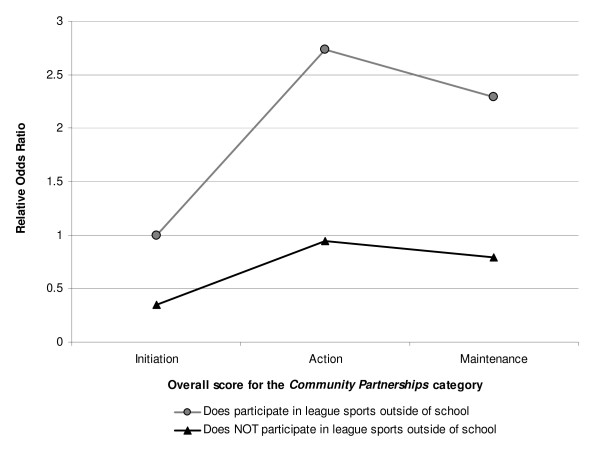
**Model-based estimated odds ratio for student being highly active versus low active as a function of the overall score for the Community Partnerships category at a school and whether or not the student participates in league sports outside of school**. Using the model estimates, the odds of a student being highly active can be estimated as a function of both the overall score for the *Community Partnerships *category and whether or not the student participates in league sports outside of school. In Figure 1, the model-based odds ratios of a student being highly active relative to a hypothetical student who does participate in league sports outside of school at a hypothetical school in the initiation phase for the overall score for the *Community Partnerships *category are presented.

During the additional exploratory analyses, one significant contextual interaction between a school-level characteristic and a student-level characteristic was identified. As shown in Figure 1, it appears that attending a school that is in the action or maintenance phase for the overall category score for Community Partnerships is associated with an increased likelihood of being highly active, especially among students who participate in league sports outside of school.

## Discussion

Physical inactivity has an annual financial impact totalling ~$2.1 billion in Canada [3]. As such, developing a better understanding of the factors that inhibit or promote PA among youth populations should be a public health priority. We identified that even when controlling for individual student characteristics, the characteristics of the school a student attends were associated with his/her likelihood of being either moderately active or highly active. This finding is consistent with previous empirical research which suggests that characteristics of the school environment can have an important impact on the PA level of a student [13-15]. We also identified that the school characteristics associated with PA were not the same for differentiating youth who were moderately active from youth who were considered highly active. This suggests that a targeted approach may be required for PA promotion depending on what youth sub-populations school-based programs may be trying to impact. For instance, our results suggest that while programs using PA as a reward may increase the likelihood of students being moderately active, schools may need to implement community partnership programs if they are interested in increasing the likelihood of students being highly active. Such a targeted approach to program implementation would require evaluation. Considering that there is more evidence from secondary school settings compared to elementary school settings [17], and there is even less evidence simultaneously examining how multiple school characteristics (programs, policies and resources) are associated with PA [13], our findings provide valuable new insight to both researchers and practitioners.

Consistent with research demonstrating that community coalitions can affect youth behaviour [7,12,17,28,29], we identified that students were more likely to be highly active if they attended a school that had established community partnerships. This includes partnering with public health units, partnering with community-based recreation clubs and organizations, and providing staff with ongoing training and support [20]. Given the importance of community-based support and reinforcement on establishing effective school initiatives [11], it was promising to see that over three quarters of the schools in our Ontario sample were in the action or maintenance phase for developing community partnerships. This was substantially higher than the published results from the American Trial of Activity for Adolescent Girls study, where just over a third of schools had collaborated with groups in the community to provide students with PA programs [7]. The Action Schools! BC program provides a good model for understanding the mechanisms by which schools can collaborate with community stakeholders to promote PA among youth [30]. A survey that employed the same SHAPES school-level tool found that schools with a "healthy school committee", often including community members, was more likely to achieve a maintenance classification for offering students a healthy school environment (unpublished data).

In this study, we also identified that some students were more likely to be highly active as a function of both their individual behaviour and whether or not the school they attended had established community partnerships. As illustrated in Figure 1, although students who participate in league sports outside of school were more likely to be highly active than students who do not participate in league sports outside of school, the strength of the association appears to be moderated by community partnerships. Attending a school that was in the action or maintenance phase for community partnerships was associated with a substantially larger likelihood of being highly active for students participating in league sports and a modest increase in the likelihood of being highly active for students who do not participate in league sports outside of school relative to students who attend a school in the initiation phase for community partnerships. Considering the physical activity levels of our respondents were similar to those of a large sample of secondary school students in Ontario [9] and data from adolescents in the United States [29], this is an important finding for practitioners interested in tailoring and/or targeting PA promotion programs to consider. For instance, there may be a larger impact by targeting programs designed to enhance community partnerships to schools in need rather than tailoring programs to all schools.

To the best of our knowledge, this is the first study to identify that students were more likely to be moderately active if they attended a school that used PA as a reward and not as discipline. It makes sense that if encouraging physical activity is the goal then physical activity experiences need to be made as positive and reinforcing as possible as opposed to allowing an association between physical activity and pain or punishment to be established [31]. Additional research is required to evaluate the potential mechanisms for using PA as a reward and the impact of such novel interventions on student PA. For instance, rewarding student behaviour by providing additional supervised areas for kids to play during the school day [14] or providing additional after school programs [32] may promote active choices in students' discretionary time.

Sedentary behaviours, such as screen time, are distinct from PA and do not necessarily replace time spent being active [8,33]. This distinction is important as research suggests that the largest public health benefit with respect to PA promotion will come from having sedentary individuals become more active rather than having active individuals become more active [29]. The American Academy of Pediatrics recommends that children's total screen time be limited to no more than 1 to 2 hours of quality programming per day [34]. In alignment with these recommendations, we identified that students with three or more hours of screen time per day were less likely to be either moderately active or highly active. However, research reviews have previously concluded that there was a zero to small association between television-based screen time and PA among youth [35]. Discrepancies between our findings and their conclusions may be due to our inclusion of other sedentary screen time behaviours, such as computer use. A recent study of sedentary behaviours in Canadian adolescents reported that computer usage was associated with physical activity among males, and reading was associated with physical activity among females [36]. We recommend that future research consider the relationship between multiple screen time behaviours rather than focusing exclusively on television/video use.

Unlike research from a provincial survey of key school informants at the elementary [37] and secondary school levels [38] in Ontario, and research from the US NHANES III [39] which identified that the majority of students report that they do not play on school based sports teams, we identified that the majority of the students in our elementary school sample reported participating in sports teams at school. This is important considering that in the present study those students who participated in intramural sports at school were almost twice as likely to be considered moderately active and three times more likely to be highly active. This finding is consistent with previous research [5,6] and research highlighting that one of the most preferable methods for engaging in PA among youth is via playing sports [16]. Alleviating barriers to the provision of intramural activities may represent an ideal opportunity for schools to intervene [37]. For instance, some schools provide students with activity buses that allow them to participate in intramural sports after schools hours [7]. Research is required to evaluate if developing programs or policies to promote student participation in intramural sports at school has an impact on increasing PA levels among students.

Behavioural theories consistently highlight the important role that influential social models surrounding youth (e.g., friends) can have on their behaviour [40,41]. In general, social models can influence behaviour through modelling, through social norms, or through providing support for the behaviour [40,41]. Empirical research has also demonstrated that the behaviour of peers are associated with higher levels of physical activity among youth [5,6] and friends' influence on physical activity levels may be higher than parental influence at least for adolescents [42]. Research has rarely considered peers as a target for PA intervention studies and this issue deserves attention in promoting a school climate that values PA.

### Limitations

This study is subject to some limitations. Almost 50% of the data for BMI were missing, so we could not robustly understand the association between weight status and PA in this sample. Since no data on ethnicity or socioeconomic status are available within our measurement tools, it was not possible to examine how PA varied across ethnic groups or social economic strata. Our ecological data were from the school environment, and it is possible that characteristics from other ecological contexts (e.g., home) may also be important to consider. Causal relationships can not be inferred from these cross-sectional data. Considering that these data were drawn from a convenience sample of schools, we can not infer that these results would be representative of the general student population in Ontario. Although data were based on self-reports, the measures in the PAM have been previously demonstrated to be reliable and valid [18], and honest reporting was encouraged by ensuring confidentiality during data collection. However, by using a measure of physical activity based on energy expenditure, we have not provided information regarding the frequency, duration or intensity of physical activity which may also be important details relevant to practitioners [43].

## Conclusion

Developing a better understanding of the school- and student-level characteristics associated with PA among youth is critical for informing intervention programs and policies designed to promote PA among youth populations. We identified that even when controlling for individual student characteristics, the characteristics of the school a student attends were associated with his/her likelihood of being either moderately active or highly active. Moreover, youth in our sample were more likely to be highly active if they attended a school with established community partnerships. Future research should evaluate if the optimal population level impact for school-based PA promotion programming might be achieved most economically if intervention selectively targeted the schools that are putting students at the greatest risk for inactivity; that is, schools that are in the initiation phase for the FHS indicators measured in the SHES tools.

## Competing interests

The authors declare that they have no competing interests.

## Authors' contributions

SL conceptualized the research question, preformed the analysis, interpretation of the results and the writing of the manuscript. SM, GF and KA helped with the interpretation of results and writing of the manuscript. CB managed the PLAY-On project and contributed to the writing of the manuscript.
